# Estimating Pesticide Exposure from Dietary Intake and Organic Food Choices: The Multi-Ethnic Study of Atherosclerosis (MESA)

**DOI:** 10.1289/ehp.1408197

**Published:** 2015-02-05

**Authors:** Cynthia L. Curl, Shirley A.A. Beresford, Richard A. Fenske, Annette L. Fitzpatrick, Chensheng Lu, Jennifer A. Nettleton, Joel D. Kaufman

**Affiliations:** 1 Department of Environmental and Occupational Health Sciences, and; 2 Department of Epidemiology, University of Washington, Seattle, Washington, USA; 3 Department of Environmental Health, Harvard University, Boston, Massachusetts, USA; 4 Department of Epidemiology, Human Genetics, and Environmental Sciences, University of Texas Health Science Center, Houston, Texas, USA; 5 Department of Medicine, University of Washington, Seattle, Washington, USA

## Abstract

**Background:**

Organophosphate pesticide (OP) exposure to the U.S. population is dominated by dietary intake. The magnitude of exposure from diet depends partly on personal decisions such as which foods to eat and whether to choose organic food. Most studies of OP exposure rely on urinary biomarkers, which are limited by short half-lives and often lack specificity to parent compounds. A reliable means of estimating long-term dietary exposure to individual OPs is needed to assess the potential relationship with adverse health effects.

**Objectives:**

We assessed long-term dietary exposure to 14 OPs among 4,466 participants in the Multi-Ethnic Study of Atherosclerosis, and examined the influence of organic produce consumption on this exposure.

**Methods:**

Individual-level exposure was estimated by combining information on typical intake of specific food items with average OP residue levels on those items. In an analysis restricted to a subset of participants who reported rarely or never eating organic produce (“conventional consumers”), we assessed urinary dialkylphosphate (DAP) levels across tertiles of estimated exposure (*n* = 480). In a second analysis, we compared DAP levels across subgroups with differing self-reported organic produce consumption habits (*n* = 240).

**Results:**

Among conventional consumers, increasing tertile of estimated dietary OP exposure was associated with higher DAP concentrations (*p* < 0.05). DAP concentrations were also significantly lower in groups reporting more frequent consumption of organic produce (*p* < 0.02).

**Conclusions:**

Long-term dietary exposure to OPs was estimated from dietary intake data, and estimates were consistent with DAP measurements. More frequent consumption of organic produce was associated with lower DAPs.

**Citation:**

Curl CL, Beresford SA, Fenske RA, Fitzpatrick AL, Lu C, Nettleton JA, Kaufman JD. 2015. Estimating pesticide exposure from dietary intake and organic food choices: the Multi-Ethnic Study of Atherosclerosis (MESA). Environ Health Perspect 123:475–483; http://dx.doi.org/10.1289/ehp.1408197

## Introduction

Organophosphate pesticides (OPs) have been the most commonly used insecticides in the United States for more than three decades. After the passage of the Food Quality Protection Act of 1996, which required food tolerance decisions to consider cumulative and aggregate risk (Food Quality Protection Act 1996), the U.S. Environmental Protection Agency (EPA) conducted chemical-specific risk reassessments of all OPs. These reassessments resulted in substantial reductions in OP use, including the elimination of many agricultural and nearly all residential uses of OPs (Clune et al. 2012). Despite these reductions, OPs remain the primary form of insect control in American agriculture, with > 33 million pounds applied in 2007 (Grube et al. 2011). According to data from the National Health and Nutrition Examination Survey (NHANES) from 2003 to 2004, OP exposure is prevalent; metabolites of OPs were detected in the urine of > 75% of the U.S. population (Barr et al. 2011).

The U.S. EPA’s 2006 Cumulative Risk Assessment for OPs determined that the primary route of exposure in the general U.S. population is through diet (U.S. EPA 2006). Studies show that consumption of an organic diet—consisting of food grown without the use of most synthetic pesticides, including OPs—can lead to a substantial and immediate reduction in OP exposure, with metabolite levels dropping below limits of detection immediately after the introduction of organic diets (Lu et al. 2006, 2008). Concentrations of urinary OP metabolites in children consuming organic diets are consistently below limits of detection (Curl et al. 2003; Lu et al. 2006, 2008).

Many studies of OP exposure have used urinary biomarkers to estimate dose. However, OP biomarkers have significant limitations as exposure assessment tools. OP metabolites have short half-lives, only representing exposures over approximately 2 days prior to sample collection (Garfitt et al. 2002; Griffin et al. 1999; Kwong 2002), and within-individual measurements are highly variable (Attfield et al. 2014; Griffith et al. 2011; Kissel et al. 2005). Further, OP metabolites can be found, preformed, in food items and in the environment (Lu et al. 2005; Quirós-Alcalá et al. 2012; Zhang X et al. 2008). If these metabolites are excreted unchanged, as has been shown in experimental studies (Forsberg et al. 2011; Timchalk et al. 2007), exposures based on urinary biomarker levels may be overestimated. Dialkylphosphate metabolites (DAPs) are common by-products of the metabolism of most OPs and are frequently used as OP biomarkers; as described by Bravo et al. (2004), six DAPs can be used to represent combined exposure to at least 28 OPs. Because individual OPs can vary in toxicity by as much as 6,000-fold (U.S. EPA 2006), this lack of specificity limits the utility of DAPs in risk assessment. For all of these reasons, the oft-used urinary biomarkers do not provide a gold standard for OP exposure assessment, particularly for estimation of long-term exposure. A better measure is one that would accurately quantify exposure to specific parent compounds of known toxicity and would reflect typical, rather than acute, exposures.

We assessed long-term dietary OP exposure in a cohort of 4,466 participants by combining self-reported information on typical dietary intake with average residue levels in those items from a national database. We further assessed the relationship between these estimates and urinary DAP concentrations in a subset of participants with conventional diets (*n* = 480). This analysis of intermethod comparability was intended as a check on the face validity of our estimates. In a second subset of participants (*n* = 240), we investigated the association between self-reported organic produce consumption habits and urinary DAP levels.

## Methods

*Study population*. The Multi-Ethnic Study of Atherosclerosis (MESA) was initiated in 1999 to investigate the progression of subclinical cardiovascular disease among 6,814 participants from six metropolitan areas: Baltimore, Maryland; Chicago, Illinois; Forsyth County, North Carolina; Los Angeles County, California; New York, New York; and St. Paul, Minnesota (Bild et al. 2002). Participants were recruited using random-digit dialing and mailed brochures, and were 45–84 years of age at enrollment with an approximately equal gender ratio. The MESA cohort is 39% Caucasian, 28% African American, 22% Hispanic, and 12% Chinese American, and all participants were free of clinical cardiovascular disease at recruitment. MESA was approved by the institutional review boards at the universities where they were recruited, and all subjects gave written informed consent.

*MESA Food Frequency Questionnaire (FFQ)*. Most data collection in MESA is structured around a series of clinical examinations, scheduled at approximately 2-year intervals. The analysis presented here employs data collected at the most recent exam, “Exam 5,” which spanned April 2010 through February 2012. All participants attending this exam were asked to complete a modified Block-style 120-item FFQ, in which they were asked about their “usual” consumption frequency and serving size of specific foods and beverages “over the past year.” Characteristic of the Block FFQ designs, serving sizes were quantified as small, medium, or large. The MESA FFQ was developed for a multi-ethnic population, and the validity of this tool has been demonstrated previously (Nettleton et al. 2009).

The MESA FFQ includes 20 line items pertaining specifically to fruits and vegetables, with each line item referring to between one and six individual foods. This study included any fruit or vegetable for which there was both intake data available from the FFQ and pesticide residue data from the U.S. Department of Agriculture (USDA) Pesticide Data Program (PDP). The following foods were included in this analysis: apples, apple juice, asparagus, blueberries, broccoli, cantaloupe, grapes, green beans, collard greens, lettuce, mangoes, nectarines, oranges, peaches, pears, spinach, strawberries, summer squash, sweet potatoes, and tomatoes. Fruit and vegetable components of mixed dishes were not considered.

Data from the Nutrition Data System for Research (NDS-R database; Nutrition Coordinating Center, Minneapolis, MN)was used to estimate the relative frequency of consumption of each food within a line item. For example, if a participant reported eating one medium serving per day of “Apples, applesauce, and pears,” they were assumed to consume 0.86 servings of apples, 0.08 servings of applesauce, and 0.06 servings of pears each day. Gram weights per serving were imputed according to survey data from NHANES (USDA 2006).

In addition to questions about food and beverage intake, participants were also asked about consumption of organic produce. Specifically, participants were asked how often the fruit and vegetables they ate were organically grown, defined as “[having] a ‘USDA Organic’ label, purchased locally from an ‘organic farm’, or grown without pesticides in a home garden.” Options were “Seldom or never,” “Sometimes,” and “Often or always.” This study included all participants who completed an Exam 5 MESA FFQ.

*The USDA PDP*. Comprehensive information on pesticide residues in food at “point-of-sale” locations (e.g., grocery stores) is provided by the USDA through their Pesticide Data Program (USDA 2014). Since 1991, the PDP has repeatedly tested > 95 commodities, including fruits, vegetables, and juices, for residues of > 450 pesticides, including all OPs registered in the United States or for which there are import tolerances. Each year, nearly 2 million analyses are conducted through the PDP, and the results are publically available (USDA 2014). PDP samples are selected without regard to country of origin, variety, or organic labeling, although relatively few samples included in the PDP database are organic (e.g., in 2010, < 3% of all included samples carried an organic label) (USDA 2012). Therefore, we treated the PDP data as if it represented conventionally grown produce.

Not all commodities are monitored in every year. To capture a more complete set of food items to which OPs are applied, we combined PDP data from 2008 through 2010. We included all OP pesticides that were detected at least once in a fruit or vegetable that was also listed on the MESA FFQ. This resulted in inclusion of the following 14 OPs: azinphosmethyl, chlorpyrifos, diazinon, dichlorovos, dimethoate, malathion, methidathion, omethoate, oxydemeton methyl, phosmet, acephate, bensulide, ethoprop, and methamidophos.

*Dietary exposure assessment: food consumption–chemical residue (FCCR) approach*. After identifying the specific pesticides and food items to be included in this analysis, we calculated the average concentration of each OP measured in each food item. PDP samples with values below detection limits were set to zero. To calculate dietary OP exposure, we combined individual-level intake data from FFQs and average residue data from the PDP using an FCCR approach (MacIntosh et al. 2001). These estimates were calculated exclusively based on food intake information and did not incorporate information on self-reported organic consumption habits.

Individual-level exposures were calculated in two ways. First, we calculated exposure in units of methamidophos equivalents to provide a metric that can inform risk assessment. We multiplied each individual’s typical intake of each food item by the average residue of each OP measured on that food. The result was then multiplied by the relative toxicity of that particular OP compared with an index chemical (methamidophos), using a relative potency factor approach (U.S. EPA 2006). This generated exposures that were then summed across pesticides and food items to yield an estimate of total daily exposure for each participant. This value was then divided by the participant’s body weight. The equation is shown below:

Exposure [ng methamidophos equivalents ÷ (kg body weight × day)] = [average daily intake (g food/day) × concentration (ng OP/g food) × toxicity (unitless)] ÷ body weight (kg).

These “methamidophos-equivalent” exposures are most useful for understanding toxicity and predicting risk, but they are not directly comparable with results from urinary biomarker analyses because the molar quantities excreted are independent of toxicity weighting and are not affected by body weight. For comparison with measurements of urinary DAPs, we calculated individual-level exposure in units of nanomoles per day. Here, we converted average OP residue levels in each food item to their molar equivalents, and multiplied that quantity by each individual’s reported typical intake of each food item:

Exposure (nmol OP/day)

= [average daily intake (g food/day) × concentration (ng OP/g food) × molecular weight (nmol OP/ng OP)].

We then summed across food items and OPs. For this analysis, we excluded four OPs that do not metabolize to form DAPs: acephate, bensulide, ethoprop, and methamidophos.

*Evaluation of FCCR-based estimates*. We did not hypothesize a strong correlation at the individual level between the FCCR-based exposure estimates and urinary biomarker measurements because of the temporal mismatch between the exposures that these measures represent (short- vs. long-term). Instead, we hypothesized that individuals with higher estimated exposures would, in aggregate, have higher DAP concentrations in any given spot urine sample than those with lower estimated exposures. Testing this hypothesis evaluates the intermethod comparability of the FCCR-based exposure estimates with the DAP measurements and thus provides a check of the face validity of our method. Because consumption of organic food has been shown to reduce OP exposure (Lu et al. 2006, 2008), we evaluated the relationship between the FCCR-based exposure estimates (in units of nanomols of OPs per day) and urinary DAP concentrations exclusively in those participants who reported that they rarely or never consumed organic produce, termed “conventional consumers.”

After calculating FCCR-based exposure estimates for each MESA participant, we evaluated the range of resulting exposure estimates among the conventional consumers. We then determined the boundaries of three tertiles of exposure—high, medium, and low—each designed to include an equal number of MESA participants. Each participant was then assigned to the appropriate tertile based on their estimated exposure. We then analyzed urinary DAP concentrations in urine samples collected from two subsets of conventional consumers, each of which included 240 participants:

Random sample. This comparison included three groups of 80 participants who were randomly selected from among each of the three tertiles of exposure.

Demographically matched sample. This comparison included three groups of 80 participants who were selected from each tertile of exposure estimates. These groups were intentionally selected to have similar distributions of gender, race/ethnicity, age, income, and education.

*Evaluation of the impact of organic consumption habits*. We were also interested in understanding the influence of self-reported organic produce consumption habits on urinary DAP levels. For this analysis, we selected 80 sets of three participants. Each set contained one participant from each category of self-reported organic produce consumption: “rarely or never,” “sometimes,” or “often or always.” These sets were matched on estimated exposure, such that the standard deviation of the exposure estimates was < 0.5 nmol OP/day within a given set. By matching on estimated exposure (by definition, a weighted metric of produce intake), we ensured that any differences in DAP concentrations were based exclusively on differences in organic consumption habits rather than on differences in produce intake. We then compared urinary DAP levels across the resulting three groups. The selected sets were constructed separately from the subsets of 240 participants described above.

*Urinary DAP biomarker analysis*. Spot urine samples were collected from all MESA participants upon arrival at the clinic for Exam 5. Samples were frozen in multiple aliquots, shipped frozen, and stored at –80°C at a central laboratory at the University of Vermont (Burlington, VT).

For this study, aliquots (1.0 mL) of selected samples were shipped frozen to the Exposure Biology Laboratory at Harvard School of Public Health. These samples were analyzed for four DAP metabolites [dimethyl-phosphate (DMP), dimethylthiophosphate (DMTP), diethylphosphate (DEP), and diethylthiophosphate (DETP)] using the method described by De Alwis et al. (2009), which involves automated solid phase extraction, on-support derivatization, and isotope dilution gas chromatography/mass spectrometry. Two DAPs [dimethyldithiophosphate (DMDTP) and diethyldithiophosphate (DEDTP)] were not measured because of analytical expense and the fact that these compounds are typically found in relatively low concentrations compared with the other DAPs (Barr et al. 2011). Quality control was assessed using standards, blanks, and spiked samples. Concentrations in laboratory blanks were all below limits of detection; because average matrix spike recoveries were high [ranging from 92% (DMP) to 105% (DETP)], samples were not recovery adjusted.

Analysis was performed in two batches, and detection limits varied between the batches: DMP, 0.5 and 1.0 ng/mL; DEP, 0.5 ng/mL in both sets; DMTP, 2.0 and 0.5 ng/mL; and DETP, 0.5 and 1.0 ng/mL. To avoid batch-related biases, samples of a given metabolite were censored at the higher detection limit. All results below the higher detection limits were assigned a value of that detection limit divided by the square root of two, a commonly applied method for substitution of censored data when the data are not expected to be normally distributed (Hornung and Reed 1990). This method is consistent with treatment of values below the limit of detection used in the Centers for Disease Control and Prevention (CDC) *Fourth National Report on Human Exposure to Environmental Chemicals* (CDC 2009).

Urinary creatinine concentration was measured via a colorimetric thin film method using the Vitros 950IRC analyzer (Johnson & Johnson Clinical Diagnostics Inc., Rochester, NY). Urinary metabolite concentrations were creatinine adjusted to account for dilution.

*Statistical analysis*. Differences in participant demographic and socioeconomic characteristics were examined across comparison groups using chi-square tests. Urinary DAP concentrations were compared across groups using generalized linear regression models, with DAP concentration as the dependent variable and comparison group as the independent variable. Post hoc Tukey HSD (honest significant difference) tests were conducted to evaluate pairwise differences in mean DAP levels across tertiles. All analyses were conducted using SAS 9.3 software (SAS Institute Inc., Cary, NC).

## Results

A total of 4,466 MESA participants attended clinic Exam 5, completed the MESA FFQ, and provided relevant demographic information (Table 1). Consistent with the MESA study design, this was a diverse group; 41% of the participants were Caucasian, 12% were Chinese American, 26% were African American, and 22% were Hispanic. The gender ratio was fairly equal, with slightly more women (53%) than men (47%). MESA is a cohort of older adults, with just over a third of this group 55–64 years of age, another third 65–74 years, and the remainder ≥ 75 years of age. Among the cohort as a whole, participants who reported more frequent consumption of organic produce also reported eating more produce overall. Median produce consumption among individuals who reported that they “often or always” ate organic produce was 3.7 servings/day, compared with 3.0 servings/day among those who “sometimes” ate organic produce and 2.2 servings/day among those who “rarely or never” did so (*p* < 0.0001).

**Table 1:** Demographic distributions (%) of all MESA participants completing an FFQ at Exam 5 (*n* = 4,466) and of subgroups based on self-report of organic produce consumption habits

	*n*	Gender		Race/ethnicity				Age (years)			Annual household income (in thousands of $US)^*a*^			Education^*b*^		
		Female	Male	White	Chinese	Black	Hispanic	< 65	65–74	≥ 75	< 30	30–75	> 75	≤ High school	Some college	≥ Bachelor’s degree
Full cohort	4,466	53	47	41	12	26	22	35	32	33	33	39	28	31	29	39
Organic produce consumption
“Rarely or never”	2,670	51	49	40	12	26	23	31	31	38	37	40	23	37	28	35
“Sometimes”	1,574	55	45	43	11	27	19	39	33	27	27	38	35	23	32	45
“Often or always”	222	65	35	39	10	23	27	44	34	23	34	35	32	24	29	48
^***a***^Information on income was missing for 147 participants. ^***b***^Information on education was missing for 7 participants.																

We estimated FCCR-based exposure estimates in units of milligram methamidophos equivalents per kilogram body weight per day (mg/kg-day) for all of these participants (Table 2). These values were lognormally distributed, with a median exposure of 2.8 ng/kg-day and an interquartile range of 1.4–5.2 ng/kg-day.

**Table 2:** Percentiles of FCCR-based exposure estimates (ng methamidophos equivalents/kg body weight-day) for all participants completing the Exam 5 FFQ and for subgroups based on self-report of organic produce consumption habits.

	*n*	Percentile of FCCR-based exposure estimates^*a*^				
		10th	25th	50th	75th	90th
Full cohort	4,466	0.69	1.4	2.8	5.2	8.6
Organic produce consumption
“Rarely or never”	2,670	0.57	1.2	2.4	4.6	7.8
“Sometimes”	1,574	0.93	1.8	3.4	5.7	9.4
“Often or always”	222	1.5	2.3	4.0	6.8	11.0
^***a***^These exposure estimates do not incorporate information on organic consumption habits; they are based exclusively on self-reported produce intake and residue levels in foods. The higher exposure estimates among individuals reporting that they “often or always” consume organic food is reflective of the fact that this group eats more produce than those who eat organic food less frequently or not at all.						

**Figure 1:**
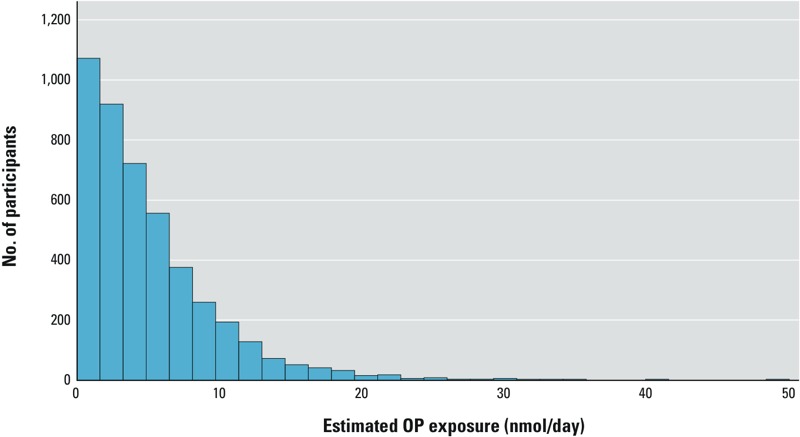
Distribution of exposure estimates (nmol OPs/day) in the MESA population (*n *= 4,466). This distribution ranges from 0 to 49.3 nmol/day (median, 3.8; interquartile range, 1.7–6.9). For analyses occurring within the subset of participants who reported they rarely or never consume organic food (*n *= 2,670), the tertiles of exposure were demarcated as follows: Tertile 1, < 1.8 nmol/day; tertile 2, 1.8–4.7 nmol/day; tertile 3, > 4.7 nmol/day.

*FCCR-based exposure estimates for comparison with urinary DAPs*. FCCR-based exposure estimates were calculated in units of nanomoles per day for all study participants; the distribution of these estimates is presented in Figure 1. This distribution is highly skewed, with a range of 0–49.3 nmol/day, a median of 3.8 nmol/day, and an interquartile range of 1.7–6.9 nmol/day.

In the analysis of the relationship between FCCR-based exposure estimates and urinary metabolite levels, we considered only those participants who reported that they rarely or never ate either organic fruit or organic vegetables (*n* = 2,670; 60%). Among these participants, the lowest tertile of exposure estimates ranged from 0 to 1.8 nmol OPs/day; the middle tertile ranged from 1.8 to 4.7 nmol OPs/day; and the highest tertile ranged from 4.7 to 49.3 nmol OPs/day (Table 3).

**Table 3:** Demographic distributions (%) of participants who were selected for urinary metabolite analysis.

	*n*	Gender		Race/ethnicity				Age (years)			Annual household income (in thousands of $US)			Education		
		Female	Male	White	Chinese	Black	Hispanic	< 65	65–74	≥ 75	< 30	30–75	> 75	≤ High school	Some college	≥ Bachelor’s degree
Subgroups selected for urinary metabolite comparison, conventional consumers^*a*^
Random sample^*b*^
Tertile 1	80	38	63	45	9	26	20	35	38	28	28	38	35	35	28	38
Tertile 2	79^*c*^	48	52	51	18	16	15	25	37	38	41	30	29	29	32	39
Tertile 3	80	59	41	56	8	20	16	28	30	43	26	43	31	21	28	51
Demographically matched sample^*d*^
Tertile 1	80	55	45	56	15	14	15	31	41	28	39	34	28	30	28	43
Tertile 2	80	56	44	55	16	14	15	31	41	28	39	34	28	30	28	43
Tertile 3	80	56	44	56	16	14	14	31	43	27	38	35	28	30	28	43
Subgroups selected for urinary metabolite comparison, by organic produce consumption habits
“Rarely or never”	80	60	40	53	9	26	13	31	36	33	21	46	33	24	25	51
“Sometimes”	80	66	34	43	9	21	28	40	35	25	26	48	26	25	33	43
“Often or always”	80	66	34	45	13	20	23	43	34	24	29	38	34	20	26	54
^***a***^Comparisons among conventional consumers are across tertiles of estimated dietary exposure to OPs. The lowest tertile (tertile 1) includes individuals with estimated exposures of < 1.8 nmol/day; the middle tertile (tertile 2) includes individuals with estimated exposures ranging from 1.8 to 4.7 nmol/day; and the highest tertile (tertile 3) includes individuals with estimated exposures > 4.7 nmol/day. ^***b***^Eighty participants were randomly selected from each tertile of predicted exposure. ^***c***^One participant was excluded because of an implausibly high urinary DAP measurement (> 30,000 nmol DAP/g creatinine). ^***d***^Participants were selected to provide three groups of 80 participants with similar frequencies of each demographic characteristic shown.																

**Figure 2:**
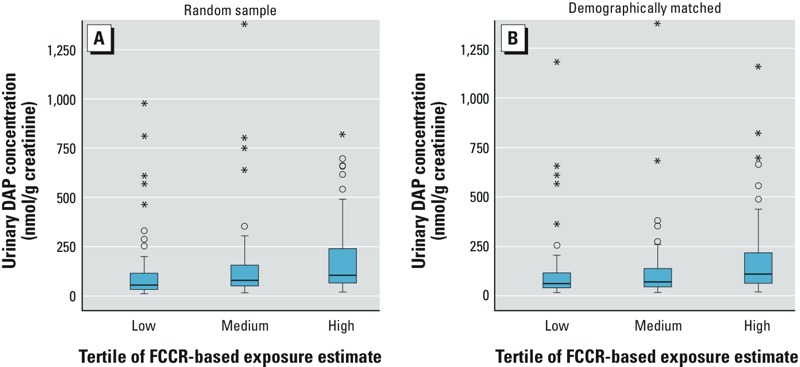
Creatinine-adjusted urinary DAP concentrations by tertile of FCCR-based exposure estimates. Boxes extend from the 25th to the 75th percentile, horizontal bars represent the median, whiskers extend 1.5 times the length of the interquartile range (IQR) above and below the 75th and 25th percentiles, respectively, and outliers are represented as asterisks (*). (*A*) This “random sample” comparison includes 80 participants randomly selected from each tertile of FCCR-based exposure estimates. One participant in the second tertile was excluded because of an implausibly high urinary DAP concentration (> 30,000 nmol DAP/g creatinine). Urinary DAP concentrations were significantly different across the three groups (*p *< 0.04). (*B*) This “demographically matched” comparison includes 80 participants from each tertile, who were selected to provide groups with similar age, gender, race/ethnicity, income, and education distributions. Urinary DAP concentrations were significantly different across the three groups (*p *< 0.03). One outlier (2,017 nmol DAP/g creatinine), which was in the “high estimated exposure” group, is not shown to preserve scale.

The first comparison of the FCCR-based exposure estimates and urinary DAP levels included three groups of 80 conventional consumers who were randomly selected from each tertile of estimated exposure to OPs. One individual who was randomly selected from tertile 2 was excluded from all analyses because of an implausibly high DAP result (33,145 nmol DAPs/g creatinine). As shown in Table 3, the lowest tertile of exposure included more men, African-American and Hispanic participants, younger individuals, and those with less education than did the higher tertiles, although this difference was statistically significant only for gender (*p* = 0.02). Table 4 shows the distributions of exposure predictions in each group included in the urinary DAP comparisons. By design, the three groups compared in the random sample have distinctly different magnitudes of estimated exposure to OPs, and urinary DAP concentrations were significantly different across groups (medians: 56, 79, and 104 nmol DAP/g creatinine, *p* < 0.04; Table 5 and Figure 2A). Post hoc pairwise comparisons of the mean DAP levels in these three groups showed the highest and lowest tertiles to be significantly different from one another; the middle group was not significantly different from the highest or lowest tertile.

**Table 4:** Percentiles of FCCR-based exposure estimates (nmol OPs/day) for participants who were selected for urinary metabolite analysis.

	*n*	Percentile of FCCR-based exposure estimates				
		10th	25th	50th	75th	90th
Subgroups selected for urinary metabolite comparison, conventional consumers^*a*^
Random sample^*b*^
Tertile 1	80	0.3	0.5	1.0	1.5	1.7
Tertile 2	79^*c*^	2.1	2.4	3.2	3.9	4.6
Tertile 3	80	5.2	6.0	7.5	10.7	13.0
Demographically matched sample^*d*^
Tertile 1	80	0.5	0.9	1.1	1.6	1.7
Tertile 2	80	2.3	2.5	3.2	4.0	4.6
Tertile 3	80	5.5	5.9	7.2	9.4	12.3
Subgroups selected for urinary metabolite comparison, by organic produce consumption habits^*e*^
“Rarely or never”	80	5.9	6.9	9.1	11.3	13.8
“Sometimes”	80	6.0	7.0	9.0	11.4	13.8
“Often or always”	80	6.1	6.9	8.9	11.6	13.8
^***a***^Comparisons among conventional consumers are across tertiles of estimated dietary exposure to OPs. The lowest tertile (tertile 1) includes individuals with estimated exposures of < 1.8 nmol/day; the middle tertile (tertile 2) includes individuals with estimated exposures ranging from 1.8 to 4.7 nmol/day; and the highest tertile (tertile 3) includes individuals with estimated exposures > 4.7 nmol/day. ^***b***^Eighty participants were randomly selected from each tertile of predicted exposure. ^***c***^One participant was excluded because of an implausibly high urinary DAP measurement (> 30,000 nmol DAP/g creatinine). ^***d***^Participants were selected to provide three groups of 80 participants with similar frequencies of relevant demographic characteristics. ^***e***^Participants were selected to provide three groups who were intentionally matched on FCCR-based exposure estimate (a metric of produce intake weighted by frequency and magnitude of OP residues detected in each food item). This is reflected in the similar values across the percentiles of exposure.						

**Table 5:** Percentiles of urinary DAP concentrations (nmol DAPs/g creatinine) by tertile of FCCR-based exposure estimates among conventional consumers and by self-report of organic produce consumption frequency.

	*n*	Percentile of urinary DAP concentration^*a*^				
		10th	25th	50th	75th	90th
Subgroups selected for urinary metabolite comparison, conventional consumers
Random sample
Tertile 1	80	24	33	56	115	228
Tertile 2	79^*b*^	33	48	79	158	275
Tertile 3	80	36	67	104	241	489
Demographically matched sample
Tertile 1	80	29	42	63	115	197
Tertile 2	80	31	47	70	137	225
Tertile 3	80	40	63	110	217	414
Subgroups selected for urinary metabolite comparison, by organic produce consumption habits
“Rarely or never”	80	48	80	163	365	638
“Sometimes”	80	39	58	121	237	474
“Often or always”	80	36	54	106	204	321
^***a***^DAP detection frequencies were as follows: DMP, 80%; DEP, 73%; DMTP, 51%; and DETP, 16%. At least one DAP was detected in 93% of the samples analyzed. ^***b***^One participant was excluded because of an implausibly high urinary DAP measurement (> 30,000 nmol DAP/g creatinine).						

The second analysis included a different three groups of 80 conventional consumers who were selected from each tertile of exposure estimates to provide each group with similar frequency distributions of gender, race/ethnicity, age, income, and education. The resulting groups were 55–56% women, 55–56% Caucasian, 15–16% Chinese, 14% African American, and 14–15% Hispanic, and similarly matched in age group, income, and education (Table 3). This demographically matched analysis yielded the same result as the random selection analysis: Urinary DAP concentrations were significantly different across the three tertiles of estimated OP exposure (medians: 63, 70, and 110 nmol DAP/g creatinine, *p* < 0.03; Table 5 and Figure 2B). As with the random sample selection, post hoc pairwise comparisons of the mean DAP levels in these three groups showed the highest and lowest tertiles to be significantly different from one another, but the middle group was not significantly different from the highest or lowest tertile.

*Evaluation of the impact of organic consumption habits*. We conducted a separate analysis of the association between urinary DAP concentration and organic consumption habits among three groups matched on FCCR-based exposure estimates but differing by self-reported frequency of organic produce consumption. Among participants included in this analysis, the median FCCR-based exposure estimate was 9.0 nmol OPs/day (interquartile range, 7.0–11.4 nmol OPs/day; Table 4). Because participants in each group were constrained to essentially match on fruit and vegetable intake (which is notably high among individuals who choose to consume organic food), the characteristics of each group are more similar than might otherwise be expected. Participants in this comparison were significantly more likely to be women (*p* < 0.001), to have a Bachelor’s degree or higher (*p* < 0.01), and to have an income greater than $30,000/year (*p* = 0.02), and were somewhat more likely to be Caucasian (*p* = 0.06) than the rest of the cohort (Table 3).

**Figure 3:**
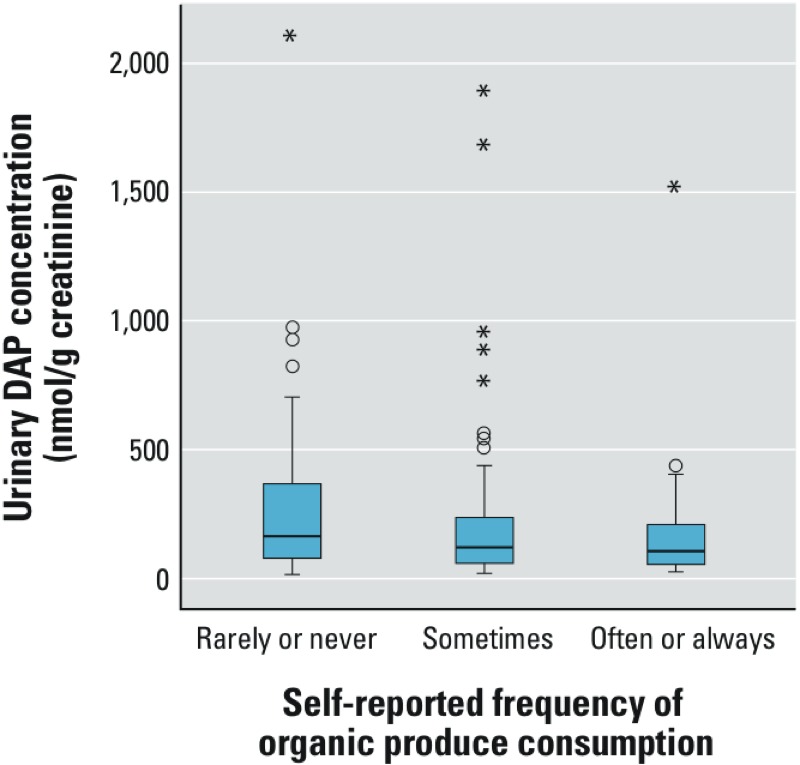
Creatinine-adjusted urinary DAP concentrations by self-report frequency of organic produce consumption. Boxes extend from the 25th to the 75th percentile, horizontal bars represent the medians, whiskers extend 1.5 times the length of the interquartile range above and below the 75th and 25th percentiles, respectively, and outliers are represented as asterisks (*). Urinary DAP concentrations were significantly different across groups (*p *< 0.02). Two outliers (3,187 and 3,707 nmol DAP/g creatinine), both of which were in the “rarely or never” group, are not shown in order to preserve scale.

We observed significant differences in urinary DAP concentrations based on self-reported frequency of organic produce consumption (*p* < 0.02; Table 5 and Figure 3). The median DAP concentrations among individuals who rarely or never consumed organic produce was 163 nmol DAP/g creatinine. Median DAP concentrations were 121 and 106 nmol DAP/g creatinine among those who sometimes consumed organic produce and among individuals who often or always ate organic produce, respectively.

## Discussion

This study provides estimates of long-term dietary OP exposure in a population for which information on organic food consumption is also available. The estimates were consistent with the results of urinary DAP biomonitoring, increasing our confidence in this methodology. DAP biomarkers are imperfect measures of long-term exposure, because of their short half-lives, lack of specificity to parent compounds, and potential to represent exposure to preformed metabolites (Sudakin and Stone 2011). Despite these limitations, numerous studies have successfully used DAPs to identify risk factors for OP exposure, including proximity to farmland (Lu et al. 2000), agricultural season and timing of pesticide applications (Koch et al. 2002), living with pesticide applicators (Loewenherz et al. 1997), or consuming conventional diets (Curl et al. 2003). In the present study, we used these DAP biomarkers in a novel way: to assess the face validity of our proposed exposure assessment method, which suffers from none of the aforementioned limitations of the DAPs. We found that low DAP levels were measured when exposure estimates were low and higher DAP levels were measured when exposure estimates were higher.

In addition to estimating dietary OP exposure, we also observed a significant relationship between increasing consumption of organic produce and lower DAP levels among individuals who were matched on FCCR-based exposure (essentially, a weighted metric of produce intake). This finding is consistent with previous studies showing that consumption of organic food measurably reduces OP exposure (Lu et al. 2006, 2008). We suggest that future studies on the effect of dietary OP intake on health include organic food consumption as a potential effect modifier in epidemiological models. This is perhaps increasingly important, as the prevalence of organic food consumption in the United States is on the rise. Several studies over the past decade have reported that 40–50% of individuals and households purchase organic food at least occasionally (Bellows et al. 2010; Onyango et al. 2007; Smith et al. 2009; Zepeda and Li 2007; Zhang F et al. 2008), consistent with the consumption frequency reported in MESA participants in this study (40%).

This is the first study of its kind to include information on organic food consumption habits, but it is not the first to use a “food consumption–chemical residue” approach to estimate dietary exposure. In an earlier Danish study, Jensen et al. (2003) evaluated the potential cumulative effects of exposure to OP and carbamate pesticides by combining dietary intake data from a nationwide food consumption survey with residue data from a Danish pesticide residue monitoring program. However, that study did not include any comparisons of the estimated exposures with biological monitoring data. Macintosh et al. (1996) estimated exposures to 11 contaminants, including three OPs, in 120,000 U.S. adults enrolled in the Nurses’ Health Study and the Health Professionals’ Follow Up Study. In subsequent analyses, the researchers analyzed arsenic and mercury concentrations in toenail samples collected from a subset of these participants (MacIntosh et al. 1997). Using the FCCR approach, the authors compared estimated arsenic and mercury exposures with measured levels in the toenails and found significant, although somewhat weak, correlations (Spearman correlation coefficients of 0.15 and 0.35). These coefficients are within the range previously found for similarly estimated dietary intake of chlorpyrifos and urinary 3,5,6-trichloro-2-pyridinol (MacIntosh et al. 2001), as well as chlordecone from diet and in blood (Guldner et al. 2010). These coefficients are also similar to coefficients observed between FFQ-based estimates of intake and biomarkers of dietary carotene (Russell-Briefel et al. 1985) and polyunsaturated fat (Hunter et al. 1992).

In the present study, we chose not to focus on correlation coefficients between the FCCR-based OP exposure estimates and urinary DAP metabolites. From the outset, we did not hypothesize a strong direct correlation between individual results from these assessment methods because of the temporal mismatch between the biological markers and the dietary information captured in the FFQ. The short half-lives of OP metabolites, as well as their correspondingly high intraindividual variability, are well established (Griffith et al. 2011; Kissel et al. 2005), and the purpose of our study was to develop a metric for long-term dietary OP exposure—which the DAP biomarkers simply cannot do. Therefore, we focused the DAP analysis on providing a check of the face validity of our exposure estimates rather than evaluating the direct agreement between the two assessment techniques; the results of this study suggest that our approach was successful and informative.

We estimated chronic dietary OP exposure for the MESA population in units of methamidophos equivalents per kilogram of body weight per day. Unlike urinary biomarkers, these estimates can be used to inform risk. Within the MESA cohort, the 95th percentile of exposure was 11 ng/kg-day. For comparison, the U.S. EPA’s 2006 OP Cumulative Risk Assessment estimated a 95th percentile of single-day dietary exposure of 92 ng/kg-day for adults > 50 years of age. (U.S. EPA 2006). This difference may reflect both the earlier data used in that risk assessment (prior to the more recent reductions in OP use) and the fact that the U.S. EPA was predicting a single-day maximum, whereas we are predicting typical exposure over the course of a year. Given that the U.S. EPA determined that those estimates were protective of health within an 870-fold margin of error (U.S. EPA 2006), our results do not suggest unacceptable risk using the risk benchmarks employed in that assessment, which are based on thresholds of cholinesterase inhibition.

Although levels from the present study are below current risk thresholds (U.S. EPA 2006), those thresholds may not adequately account for the potential synergistic effects of exposure to a mixture of pesticides—effects that have been observed in several recent animal studies (Laetz et al. 2009, 2013, 2014). Further, these thresholds may not reflect important mechanisms of low-level toxicity, which are only beginning to be understood. The importance of understanding chronic, low-level exposures to OPs is underscored by the results of several studies of the effects of low levels of OP exposure to infants and children. Mother–child cohort studies have found prenatal maternal urinary DAP levels to be significantly associated with attention problems and attention deficit/hyperactivity disorder in children at 5 years of age (Marks et al. 2010), poorer intellectual development at 7 years of age (Bouchard et al. 2011), and decreased cognitive development in children at 1 year of age and at 6–9 years (Engel et al. 2011). Another mother–child cohort study found that prenatal chlorpyrifos exposure, assessed using umbilical cord blood plasma, was associated with deficits in both memory and IQ at 7 years of age (Rauh et al. 2011). Mothers in these studies were thought to have either agricultural or residential OP exposure, in addition to dietary exposure.

Interestingly, a more recent study in a fourth cohort of mother–infant pairs found that higher prenatal maternal urinary DAP levels were associated with improved neurobehavioral outcomes among infants at 5 weeks of age (Yolton et al. 2013). However, the mothers with higher OP exposure also reported more frequent consumption of fruits and vegetables than those with lower OP exposure, suggesting that total produce intake (and other correlated residual confounders associated with socioeconomic status) may be critical to consider, particularly when diet is likely to be the only significant source of OP exposure.

The present study adds to the existing literature regarding the relationship between organic food consumption and DAP levels (Curl et al. 2003; Lu et al. 2006). Here, we found that—when matched on produce intake—individuals who reported eating organic produce at least occasionally had significantly lower urinary DAP levels than those who ate primarily conventional produce. This finding held only when produce intake was considered because the median DAP level among individuals who consumed organic produce was higher than the median level in the conventional consumer group as a whole. On its face, this finding is counterintuitive and perhaps even concerning because it might suggest that organic produce is not actually free of OP pesticides. However, we hypothesize that this reflects the difference in total produce consumption among these groups. In our study, we did not include a group of individuals who exclusively ate organic produce, and it is difficult to know exactly how much of a participant’s diet is organic when they report that organic produce is “often” eaten. We suspect that the conventional fraction of the total produce intake among participants who “often” consume organic produce is responsible for the DAPs present in their urine. Alternate explanations for this finding would be higher exposure to OPs (or DAPs) from environmental sources or in organic food itself, but our study includes no data to support these explanations.

Although our study provides a method for assessing long-term dietary OP exposure and supports that method with the results of urinary biomonitoring, there are several limitations. Notably, we were only able to compare these estimates to the very biomarkers we find lacking. Unfortunately, this is the state of the science: No gold standard is available. Another limitation is that these FCCR-based estimates are based on data acquired from FFQs, which can be limited by recall bias. However, among all dietary assessment tools, FFQs are best suited to provide information for studies where typical, long-term diet is the conceptually important exposure, rather than intake on a few specific days (Willett 1998). This is a strength of using FFQ data in the present study because individuals are known to do a better job of recalling their usual diets rather than describing what foods were eaten in any specific meal in the past (Willett 1998).

The PDP database provides the most comprehensive OP residue data available, but it is a national database and does not reflect the specific residues to which individuals are exposed. We also did not include every food item to which OPs are applied. Some items were excluded because no OPs were detected on any samples of that type, which is unlikely to affect the results of this study. Other items were excluded because they were not included on the MESA FFQ. We also did not include fruit and vegetable components of mixed dishes because we were concerned that this would increase uncertainty in our analyses. Future studies might benefit from including an FFQ specifically designed for pesticide exposure assessment purposes. Further, although our study benefited from the large sample size available in MESA, that study is limited to older adults. Future research on the ability of this method to estimate exposures among children and younger adults is warranted.

A final limitation of our study is that we did not allow produce consumption frequency and organic produce choice to vary freely within the analytic data sets. Because of the costs associated with determining the DAP concentration, we made some statistical selections to maximize efficiency while avoiding confounding. Among conventional consumers, we evaluated the relationship between FCCR-based exposure predictions and urinary DAP concentrations using two different sampling strategies and found consistent results. The first comparison included individuals who were randomly selected from across the spectrum of estimated exposure, removing the possibility of selection bias. However, the FCCR-based estimates are, by definition, highly correlated with produce consumption, which is in turn related to demographic and socioeconomic factors. Distributions of demographics and socioeconomics were notably different across the randomly selected groups. By evaluating DAP concentrations among groups matched on these characteristics, as in our second comparison, we can be fairly confident that the observed differences in DAPs were not related to these factors.

Although our study has limitations, it also has several notable strengths. We employed a large and well-characterized cohort for which data was collected using standardized data collection methods. The exposure estimation approach we propose allows identification of parent compounds, which in turn allows evaluation of risk. Compared with urinary metabolite analysis, the FCCR-based assessment approach is noninvasive and inexpensive, and it can be easily implemented in cohorts in which FFQ data are already available (although information on organic food consumption habits may still need to be acquired). Further, this methodology provides estimates of long-term exposure, which cannot be obtained from existing biomarker methods.

The FCCR method described here may prove useful in future epidemiological studies of long-term dietary OP exposure, particularly if it is paired with information on organic food consumption, which may modify the observed exposure–response relationship. As concern grows regarding potential effects of low-level OP exposures, the need increases for more sophisticated exposure assessment methods. These methods must take into account the relevant time frame of exposure and be able to define the parent compounds to which individuals are exposed in order to truly assess risk.
